# A Record of the Mortality of the City of Aurora, from July 1, 1858, to July 1, 1859

**Published:** 1859-08

**Authors:** L. H. Angell

**Affiliations:** Aurora


### ARTICLE V.

### A RECORD OF THE MORTALITY OF THE CITY OF AURORA, FROM JULY 1, 1858, TO JULY 1, 1859.

[Read before the Aurora Medical Association, and communicated for the *Chicago Medical Journal.*]

BY L. H. ANGELL, M.D.

During the past year I have been at some pains to keep a record of the deaths which have occurred within the city. Our town, now containing a population of eight thousand, should adopt some efficient system of registration of its mortality, which would certainly furnish statistics of great and increasing value for all future time.

As is shown by this record—which I believe, in most of its details, nearly correct—the past year with us has been one of small mortality, and will furnish some evidence to establish the reputation for salubrity of the valley of Fox river, particularly at this point. Located upon the banks of a river which furnishes sufficient water power for various mills and manufactories, it would not be surprising if malarious disease should prevail at certain seasons, to such extent as to enhance the mortality. But the river through the city limits having sufficient fall to run with considerable current has no interruption, except that occasioned by the dam. This, at the foot of Main street, leaves a small portion of the city above exposed to malaria usually generated by such cause. Aside from this there is hardly a locality within the city open to this objection. The site of the town upon either bluff being sufficiently elevated to afford facilities for drainage in the direction of the river, which has been judiciously effected where it was needed, there now exist few localities where water will be seen upon the surface, even after the most protracted rain. Prior to '56, a portion of Broadway, in the East Division, and a section just north of this, were particularly obnoxious,—the one for a collection of water and filth, and the other for being originally a broad slough; but now the former is occupied by blocks of brick buildings, and the latter by the machine and car shops of the C., B. and Q. R. R., and, of course, thoroughly renovated in every particular; so there now seems to be no reason why we should be liable to zymotic diseases from other than general or meteorological causes. Located as we are, forty-three miles west of Chicago, in the midst of prairie, with ample and wide streets and parks,—the lungs of a town,—we should be, as *we are*, second to no other in our sanitary condition, which is fully demonstrated by the following record:

*Whole number of deaths, from July* 1, 1858, *July* 1, 1859, - 57

| | |
|---|---|
| Children under 5 years, - - - | 36 |
| Between 5 and 10 " - - - - | 2 |
| " 10 " 20 " - - - - | 2 |
| " 20 " 30 " - - - - | 4 |
| " 30 " 40 " - - - - | 5 |
| " 40 " 50 " - - - - | 5 |
| " 50 " 70 " - - - - | 2 |
| " 80 " 90 " - - - - | 1 |

*Diseases of Children under 5 years.*

| | |
|---|---|
| Pneumonia, - - - - - - - - | 11 |
| Cholera Infantum, - - - - - - - | 2 |
| Scarlatina, - - - - - - - - | 3 |
| Croup, - - - - - - - - - | 2 |
| Congestion Lungs, - - - - - - - | 2 |
| Convulsions, - - - - - - - - | 3 |
| Diptheria, - - - - - - - - | 1 |
| Diarrhœa, - - - - - - - - - | 1 |
| Inflammation of Brain, - - - - - - | 1 |
| Stillborn, - - - - - - - - - | 1 |
| Unknown, - - - - - - - - - | 9 |
| | 36 |

Diseases of those upwards of five years: phthisis pulmonalis, 5; typhoid fever, 2; and 1 each of the following causes—puerperal fever, ulceration and perforation of bowels, disease of heart, scirrhus of pancreas and duodenum, pneumonia, ascites, cancer uterus, congestion of brain, cholera morbus, psoas abscess, pericarditis, accident from burn, apoplexy, and old age.

It will be seen by this that our acute affections have been generally amenable to treatment, and that the greatest mortality has occurred from pneumonia of children and infants. This affection has been quite prevalent, and I believe the cases have generally yielded to treatment when early resorted to, and before empirical remedies have had a too protracted trial, at the hands of a nurse or would-be-doctor. I will now mention another feature which this record contains which is not without interest to the profession, in estimating the curability of our affections. Of these fifty-seven (57) fatal cases, fifteen (15) occurred in the hands of regular physicians, and thirty-five (35) were patients of irregular practitioners, including the different "pathies," eclectics and advertising doctors, who practise among us. Of the fifteen (15), more than half of this number were of incurable chronic diseases, and a portion of the remainder were subjected to irregular treatment until moribund.

It is somewhat singular that twenty-seven (27) of these fatal cases should have been patients of an empiric who boasted publicly of "not ringing the bells over his patients," while the nine members of this association who are devoting their entire attention to the practice of the profession, should have, in the same time and within the same city limits, but fifteen (15) fatal cases.

In the name of "*little children*," who should "ring bells over their patients?"

AURORA, July 4, 1859.

